# Nitrogen Concentration Estimation in Tomato Leaves by VIS-NIR Non-Destructive Spectroscopy

**DOI:** 10.3390/s110606411

**Published:** 2011-06-16

**Authors:** Valentina Ulissi, Francesca Antonucci, Paolo Benincasa, Michela Farneselli, Giacomo Tosti, Marcello Guiducci, Francesco Tei, Corrado Costa, Federico Pallottino, Luigi Pari, Paolo Menesatti

**Affiliations:** 1 Department of Agricultural and Environmental Sciences, University of Perugia, Borgo XX Giugno 74, 06121, Perugia, Italy; E-Mails: valentina.ulissi@yahoo.it (V.U.); paoloben@unipg.it (P.B.); michela.farneselli@unipg.it (M.F.); giacomo.tosti@gmail.com (G.T.); mguid@unipg.it (M.G.); f.tei@unipg.it (F.T.); 2 Agricultural Engineering Research Unit of the Agricultural Research Council (CRA-ING), Via della Pascolare 16, 00015, Monterotondo scalo (Rome), Italy; E-Mails: corrado.costa@entecra.it (C.C.); fedepall@yahoo.it (F.P.); luigi.pari@entecra.it (L.P.); paolo.menesatti@entecra.it (P.M.)

**Keywords:** spectrophotometry, VIS-NIR, tomato leaf, non-destructive, SPAD chlorophyll meter readings, SAP test, leaf analysis, nutritional status, chemometry

## Abstract

Nitrogen concentration in plants is normally determined by expensive and time consuming chemical analyses. As an alternative, chlorophyll meter readings and N-NO_3_ concentration determination in petiole sap were proposed, but these assays are not always satisfactory. Spectral reflectance values of tomato leaves obtained by visible-near infrared spectrophotometry are reported to be a powerful tool for the diagnosis of plant nutritional status. The aim of the study was to evaluate the possibility and the accuracy of the estimation of tomato leaf nitrogen concentration performed through a rapid, portable and non-destructive system, in comparison with chemical standard analyses, chlorophyll meter readings and N-NO_3_ concentration in petiole sap. Mean reflectance leaf values were compared to each reference chemical value by partial least squares chemometric multivariate methods. The correlation between predicted values from spectral reflectance analysis and the observed chemical values showed in the independent test highly significant correlation coefficient (*r* = 0.94). The utilization of the proposed system, increasing efficiency, allows better knowledge of nutritional status of tomato plants, with more detailed and sharp information and on wider areas. More detailed information both in space and time is an essential tool to increase and stabilize crop quality levels and to optimize the nutrient use efficiency.

## 1. Introduction

There is an increasing effort to optimize nitrogen (N) fertilization and to improve crop N use efficiency in order to achieve high yields and limit environmental side effects related to N leaching [1]. This can be done by a fine-tuning of fertilization rate and a dynamic N management at each growth stage according to the nutritional status periodically monitored [2]. This is the case of fertigated vegetables, such as processing tomatoes, where the fertilizer rate can be split and adjusted throughout the whole growing cycle by means of a drip irrigation system [3].

Nevertheless, standard laboratory analysis of N concentration in the above-ground biomass are expensive and time consuming, especially if a rapid crop N status evaluation is required for in-season decision making procedures [4]. For this reason, quick and practical tests have been proposed, some of which are already spread among growers. Opto-electronic based techniques can strongly help to reach the previously mentioned goals, thanks to easiness of use and low costs. Two of the most common and simple of these are: the chlorophyll meter readings (e.g., SPAD-502, Minolta) and the measurements of N-NO_3_ concentration in petiole sap (SAP test).

The N nutritional state in plants may be determined indirectly by the chlorophyll concentration present in the leaves, as it is directly correlated to their N concentration [5]. The SPAD method measures the light transmittance through leaves and is based on this correlation. It has been tested with good results in several arable and vegetable crops and also in tomato [2–6]. Nevertheless, the N prediction performance of the SPAD is variable, being affected by several factors such as cultivar, environmental conditions, plant growth stage, disease and pests [6]. In addition, Monje and Bugbee [7] found that data from SPAD were closely correlated with destructive measurements of chlorophyll for leaves with chlorophyll concentrations ranging from 100 to 600 mg m^−2^, but consistently overestimated chlorophyll outside this range.

The SAP test can be measured by different tools generally correlated to the conventional laboratory analysis [8]. The most commons are: Merkoquant test strips, which react to the N-NO_3_ concentration by producing a color, the intensity of which varies directly with the concentration; an ion-specific electrode, as Horiba-Cardy Meter, which reads directly the N-NO_3_ concentration in the SAP. Several plant SAP quick test kits have been calibrated for N in many arable and vegetable crops and also in tomato [2]. The N prediction performance by SAP test is variable being affected by many cultivar and agronomical factors [2], like SPAD. In tomato cultivation, it has been found to be in agreement with the critical-N curve method [9] for the most critical period of the fertilization management [10].

Both SPAD and SAP test however seem to better indicate N deficiencies than excesses, therefore their use for reducing over-fertilization would be not efficacious [11]. For these reasons it is very important to consider the critical N concentration, which is the minimum in the plant required for maximum growth [12]. Tei *et al.* [9] proposed a critical N dilution curve for processing tomato which can represent the reference to evaluate if the crop is at sub-optimal (<3.72%), optimal (between 3.72% and 4.81%) or luxury (between 4.81% and 5.2%) consumption at any time of the cycle.

In this study we propose a more complex and efficient opto-electronic method for the evaluation of N nutritional status in tomato leaves. It refers to the utilization of a visible-near infrared (VIS-NIR) portable spectrophotometer representing a rapid, non-destructive, cost-effective technique [13]. The aim of this study was to evaluate the feasibility and accuracy of this method as compared to SPAD readings and SAP test and to reference standard chemical analysis.

## 2. Experimental Section

### 2.1. Data Collection

This study was carried out in 2008 at the Experimental Station of the Department of Agricultural and Environmental Sciences, in Papiano (Tiber Valley, Perugia province, Central Italy, 43°N, elev. 165 m) on a clay-loam soil. Processing tomato (*Lycopersicon esculentum* Mill., cv. PS1296) was grown in the field according to a randomized block design with three replicates where thirteen fertilisation treatments were compared, differing for application technique, N form and rate. Five of them were represented by green manures grown in the previous fall-winter and incorporated in early spring: green manures were hairy vetch (*Vicia villosa* Roth.) and barley (*Hordeum vulgare* L.) cultivated as monocultures at full sowing density (200 seeds m^−2^ for vetch and 400 seeds m^−2^ for barley) and as intercrops obtained by using a fraction of the full sowing density for each species according to a substitutional approach, namely 75% of vetch + 25% of barley, 50% + 50% and 25% + 75%. The total N supplied by green manures varied from 252 kg ha^−1^ to 183, 167, 160 and 154 as the proportion of vetch decreased from 100% (pure vetch) to 0% (pure barley). Previous experiments have shown that actual N release in the soil from green manures above is much different in time, with pure barley causing N deficiency during early stages of the following cash crop [14,15]. The other eight treatments included: broadcast all-at-once application of two organic fertilisers (poultry manure and by-product from leather factory, both at 100 kg N ha^−1^); localised and split fertigation with one organic and one mineral fertiliser at 2 different rates (100 and 200 kg N ha^−1^); two unfertilised controls, one with tomato in plots where no crop was grown in autumn-winter and one with tomato in plots where barley was grown and then mown and removed from the field before tomato transplanting in order to cause the maximum depletion of soil available N.

The supply of P and K was adjusted taking into account the amount supplied with organic fertilizers, in order to obtain the same rate for all N fertilization treatments (75 kg ha^−1^ of P_2_O_5_ and 75 kg ha^−1^ of K_2_O). The same irrigation volume was applied in a two-times-per-week irrigation schedule for all treatments, according to potential crop evapotranspiration. The N nutritional status of the crop was evaluated on three sampling periods (s.p.), 25 June (37 Days After Transplanting, DAT), 9 July (51 DAT), and 23 July (65 DAT), in coincidence with plant samplings for growth analysis (1st s.p., 2nd s.p. and 3rd s.p. respectively). Each sampling period corresponds to a specific phenologic stage of the tomato plantation: 1st vegetative growth, 2nd early flower fruit and 3rd fruit bulking.

At each sampling date eight plants per plot were harvested. The SPAD readings were taken on the apical leaflet blade of the youngest fully expanded leaf of those plants; the petioles of the same leaves were then collected and SAP nitrate concentration was measured by an ion-specific electrode meter. Then the eight leaflets above, plus other 16 leaflets detached from other young fully expanded leaves of the same eight plants per plot, were stored at 5 °C in plastic envelopes in the dark and carried to the CRA-ING laboratory (Lat. 42°06′11.00″ N, Long. 12°37′40.81″ E) where VIS-NIR measurements were performed within three hours. Thirteen-fifteen leaves were spectrally measured two times, randomly acquiring nearly 3,100 full spectra. The N concentrations of the leaflets used for VIS-NIR measurements and of the whole above-ground plant subsamples were then measured by analysis of dry matter; an automatic analyser (FlowSys, Systea, Italy) was used to measure organic-N concentrations on digests prepared according to Isaac and Johnson [16].

### 2.2. SPAD Analysis and SAP Test

The SPAD readings were taken from the apical leaflet of the youngest fully expanded leaf; the petioles of the same leaves were then collected and SAP nitrate concentration was measured by an ion-specific electrode meter (Cardy, Spectrum Technologies, Inc., Plainfield, IL, USA). The N concentration of the leaves and of the whole plant were then measured by analysis of dry matter; an automatic analyser (FlowSys, Systea, Italy) was used to measure organic-N concentrations on digests prepared according to Isaac and Johnson [16].

### 2.3. Spectrophotometric Analysis

For the VIS-NIR measurements, a (portable) single channel spectrophotometer was used. The system is composed of five parts: (1) a Hamamatsu S 3904 256Q spectrograph in a special housing; a customized illumination system realized by a 20 W halogen lamp and an optical fiber bundle consisting of approx. 30 quartz glass; (2) an optical entrance with input round: 70 μm × 2,500 μm and diameter 0.5 mm NA = 0.22 mounted in SubMiniature version A-coupling; (3) specific probes with quartz optical fiber of connection; (4) a transmission device for transmitted or absorbed light for thin solids or liquid with variable optical length; (5) a notebook equipped with specific software to acquire, calibrate and elaborate spectral data. The Hamamatsu spectrograph has the following characteristics: grating: flat-field, 366 line/mm (centre); spectral range: 310–1,100 nm; wavelength accuracy absolute: 0.3 nm; temperature—induced drift: <0.02 nm/K; resolution (Rayleigh-criterion): DlRayleigh ≫ 10 nm; sensitivity: ≫1,013 Counts/Ws (with 14-Bit-conversion); straylight: <0.8% with halogen lamp and 16 bit A/D converter.

For spectral acquisition, the ‘pen’ probe was used to measure the spectral reflectance response on each single leaf (spot area ≈ 10 mm^2^). On each leaf two spot areas were acquired with the pen probe in the same areas used for SPAD and SAP test analysis. The reflectance measure is referred to the light percentage that is reflected by the material and acquired by an optical quartz fiber (0.7 mm in diameter) fixed at 45°inside a circular aperture of 4 mm in diameter, in relation with a white reference (100% of the signal available). The material surface due to its softness was able to include the entire circular aperture avoiding any external light interference. The spectral measurements were performed in laboratory considering a white calibration (lower value with respect to the external light), the instrumental integration time (light acquisition time) and subtracting the background noise (variable in function of the instrument temperature). A very low signal/noise ratio was observed in the beginning and at the end of the spectral range, affecting the accuracy measurements, so only the spectrum in the range 400–800 nm were take into account for the analysis. All spectral values were expressed in terms of relative reflectance. To remove drift effect for each group 12–15 leaves were chosen at random. After 30–35 spectral measurements a new white calibration was performed.

### 2.4. Chemometric Analysis of Spectral Data

**Figure 1.**
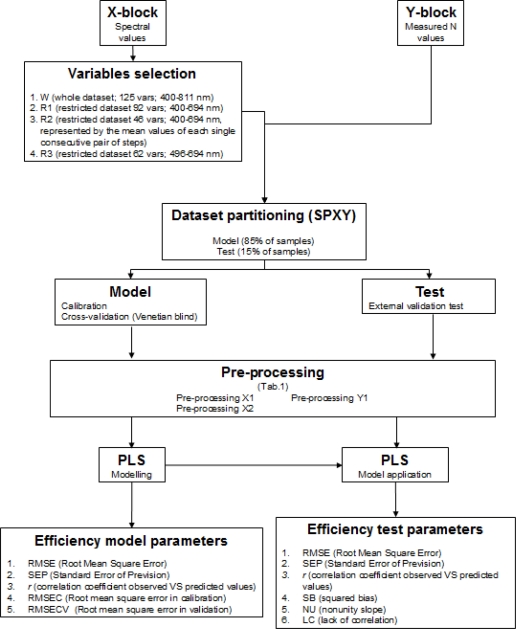
Spectral data chemometric analysis procedure.

Mean reflectance values of all leaves considering together all treatments, were compared to each reference chemical value by chemometric multivariate methods (Partial Least Squares, PLS). The procedure includes the following steps (Figure 1): (1) extraction of raw spectra dataset, to be used as X-block variables; (2) X-block variables selection; (3) creation of measured values dataset to be used as reference or response variable (Y-block); (4) data fusion of the two dataset (X- and Y-block) in one analysis dataset; (5) SPXY (sample set partitioning based on joint X- and Y-blocks) [17] partitioning of the dataset into two subsets, one for the model (85% of whole dataset) and one for the external validation test (15% of whole dataset) (*i.e.*, 85% of total samples were used to calibrate model and 15% were reserved for external validation). With respect to the random partitioning method, widely used in literature, SPXY approach, assigning equal importance to the samples distribution within both X- and Y-blocks, returns more objective and replicable results and could found analytical applications involving complex matrices, in which the composition variability of real samples cannot be easily reproduced by optimized experimental designs; (6) application of different pre-processing algorithms to X-block and Y; (7) application of chemometric technique (PLS): modelling and testing; (8) calculation of efficiency parameter of prediction.

All the spectral variables in the range 400–800 nm were selected to obtain four different X-block datasets: (i) Whole dataset (W; 125 vars; 400–811 nm); (ii) Restricted dataset 1 (R1; using the first 92 vars; 400–694 nm); (iii) Restricted dataset 2 (R2; 46 vars; 400–694 nm) represented by the mean values of each single consecutive pair of steps; (iv) Restricted dataset 3 (R3; 62 vars; 496–694 nm).

To divide the dataset into model (calibration and validation) and test sub-sets, for multivariate PLS analysis, the SPXY method [17] was used. This method employs a partitioning algorithm that takes into account the variability in both x- and y-spaces. To obtain the best prediction test, different X and Y pre-processing techniques were applied (Table 1), from the simpler (none, Log 1/R, diff1, mean centre, autoscale, median centre, baseline) to the more specific for spectral data (Savitsky Golay, Multiple Scatter Correction, Orthogonal Signal correction). Pre-processing for X block was applied both as single pass then as double considering all possible combinations (*i.e.*, Log 1/R + autoscale).

Prediction of nitrogen leaves concentration was performed by PLS regression model, using PLS Toolbox in MATLAB V7.0 (The Math Works, Natick, MA, USA). PLS is a soft-modelling method [18] for constructing predictive models when the factors are many and highly collinear. The model works through a specific algorithm (SIMPLS) on the whole array variables (input variables, X-block) and on the observed values (Y-block), after pre-processing treatments. The model determines the minimum set of the n estimation variables (LV, latent variables) by a recursive process. These variables could be represented in a n-dimensional space and they are used by PLS to calculate the best regression matrix between the X and the Y. The calibration models were also validated using full cross-validation, Venetian blind (Matlab rel. 7.1, PLSToolbox Eigenvector rel. 4.0). The model includes a calibration phase and a validation phase and for both phases it calculates the residual errors (Root mean square error in calibration RMSEC and validation RMSECV). Modelling methods are subjected to over-fitting: this occurs when a model is excessively complex, such as having too many parameters (LV) relative to the number of observations. Therefore, in order to avoid the over-fitting is necessary to choose the model in order to optimize the number of LV in relation with efficiency parameters.

### 2.5. Regression Analysis on SPAD and SAP Data

Different monovariate regressions (linear, power, exponential and logarithmic) were calculated between the petiole nitrate concentration measured by the SAP and the total plant and leaf N concentration measured by laboratory analysis. The same analyses were also determined between the chlorophyll concentration assessed by SPAD analysis and both the total plant and leaf N concentration measured by the lab analysis.

The linear correlations between the N concentration chemically measured of the three different sampling periods (s.p. 1st, 2nd and 3rd) and the N concentration measured through SPAD analysis were calculated. To calculate the prediction efficiency, as in chemometric analysis, the whole dataset was divided into model (calibration and validation) and test sub-sets by means of the SPXY method [17].

### 2.6. Predictive Accuracy of Models

The predictions obtained from the SPAD, SAP and PLS models in external validation subset were compared through linear regression analysis with the observed values.

Different accuracy parameters were extracted such as RMSE (Root Mean Square Error), SEP (Standard Error of Prediction) and correlation coefficient (*r*). The *r* was taken into consideration for distinguishing systematic errors and studying the correlation between the reference and predicted values. Generally, a good model should have high correlation coefficients *r*, low RMSE and SEP.

Others three parameters were calculated referring to Gauch *et al.* [19]: squared bias (SB), nonunity slope (NU) and lack of correlation (LC). In formulae (1, 2 and 3) they are defined as follow:

(1) $$SB={\left(\bar{X}-\bar{Y}\right)}^{2}$$

(2) $$NU={\left(1-b\right)}^{2}\times (\sum {\left(x-\bar{X}\right)}^{2}/N)$$

(3) $$LC={\left(1-r\right)}^{2}\times (\sum {\left(y-\bar{Y}\right)}^{2}/N)$$

where x are the model-based predicted and y the measured values respectively, *X̄* and *Ȳ* their mean, N the number of observations in the validation test, b is the slope and *r*^2^ the square of the correlation.

In a perfect prediction, *i.e.*, in the 1:1 line of equality Y = X, SB and LC should be equal to 0, while NU > 0 for b ≠ 1. In the accuracy analysis, SB is a good indicator of translation, NU of the rotation and LC of the scattering of the correlation line [19].

## 3. Results

**Figure 2.**
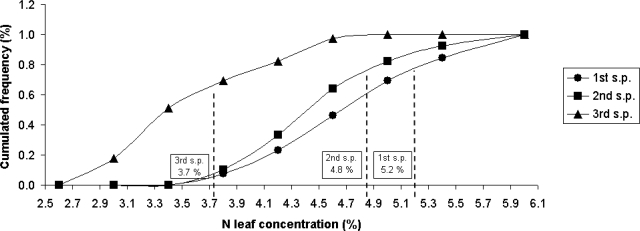
Cumulated nitrogen concentration frequency chemically measured in relation to the three different sampling periods (s.p. 1st, 2nd and 3rd) and the three thresholds (vertical lines) extracted by the critical-N curve proposed by Tei *et al.* [9].

Figure 2 shows the cumulated nitrogen concentration frequency chemically measured in relation to the three different sampling periods (s.p. 1st, 2nd and 3rd).

In the figure, the three thresholds extracted by the critical-N curve proposed by Tei *et al.* [9] are also reported. These thresholds indicate the optimum N concentration of the plants depending on the phenologic stage of the tomato plantation. Approximately 80% of the samples were below the critical threshold of N leaf concentration during the first two sampling period, and ∼65% were below the threshold for the 3rd sampling period.

The *r* values of the linear correlations between the N concentration chemically measured divided in the three different sampling periods (s.p. 1st, 2nd and 3rd) and the N concentration measured through SPAD analysis resulted very low: 0.5, 0.2 and 0.3 respectively.

Table 2 indicates the results of the linear regression of the model and test performed on the values of SPAD and SAP. The coefficient of correlation (*r*) of the test is equal to 0.56 in both SPAD and SAP test. The SEP and RMSE values are slightly lower for the SPAD analysis (0.72 and 0.64 respectively). Table 2 reports also the squared bias (SB), the nonunity slope (NU) and the lack of correlation (LC) for both SPAD and SAP test. These values are respectively equal to: SB = 0.12 and 0.12; NU = 0.0003 and 0.05; LC = 0.48 and 0.48.

**Figure 3.**
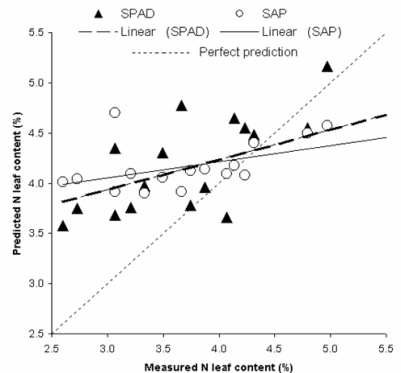
The correlation between measured and predicted values of N of SPAD (chlorophyll meter readings) and SAP (measurements of N-NO_3_ concentration in petiole) analysis in the test represented by the 15% of the whole sample dataset extracted by the SPXY (sample set partitioning based on joint X- and Y-blocks) method.

Figure 3 shows the correlation between measured and predicted values of N by SPAD and SAP analysis in the test represented by the 15% of the whole sample dataset extracted by the SPXY method. In Table 3 values and results of PLS models and test prediction on the four spectral datasets (W, R1, R2 and R3) and of N concen tration in tomato leaves from spectral reflectance analysis are reported.

The best model was the R3 using only the central values of the spectra (496–694 nm). This model uses firstly a Log1/r pre-processing on the X-block and then a snv pre-processing on the pre-processed X-block. Y-block was not pre-processed. The *r* value of the test is very high: 0.94 (Table 3).

Also the values of SEP and RMSE of the test are very low (0.35 and 0.40 respectively). The prediction ability of the model revealed to be high, being a SEP of 0.43 and the RMSE of 0.43 indicates that predictions were on average within 0.43% N of the measured values. Moreover, the values of the predictive accuracy are equal to: SB = 0.05, NU = 0.0188 and LC = 0.09.

## 4. Discussion and Conclusions

Usually N nutrition is determined by leaf chemical analysis, which presents some disadvantages that limit its use, such as the length of sampling time, the use of hand labour, the need for specialized equipment and high cost [20]. Thus, according to Guimarães *et al.* [21] alternative methods that using portable gauges, permitting diagnosis and monitoring of the N nutrition of the plants in a faster and non-destructive way in the field are required.

In this study the estimation efficiency of the N concentration of tomato leaves determined by a portable VIS-NIR spectrophotometer by means of chemometric procedures resulted always higher than these obtained by SPAD chlorophyll meter readings and SAP tests, as demonstrated for the parameters *r* (0.94 *vs.* 0.56), SEP (near 40% lower), RMSE values (near 35% lower), SB (0.05 *vs.* 0.12), NU (0.0188 for VIS-NIR *vs.* 0.0003 for SPAD and 0.0517 for SAP test) and finally LC (0.09 for VIS-NIR *vs.* 0.48 for SPAD and SAP test). The N nutritional state in plants may be determined indirectly by the chlorophyll concentration present in the leaves, as it is directly related to their N concentration. Many studies found a high correlation between N and chlorophyll, because pigments determine most spectral features between 400 nm and 700 nm [22,23]. It was confirmed in this work by proving that a restricted spectral dataset (R3 = 496–694 nm) that refers to the spectra range of the chlorophyll, highly correlated with the analysed leaf N concentration. Similar results were obtained in the study of Min *et al.* [24] where the N leaf concentration of Chinese cabbage was detected using VIS and NIR spectroscopy in combination with PLS regression producing a *r* = 0.92. The most significant wavelength correlated to chlorophyll was identified in the 710 nm, but also wavelengths near 550 and 840 nm contributed to N prediction as in our study.

Esposti *et al.* [25] reported a SPAD chlorophyll meter for the multi-parametric chemical compound concentration estimation in leaves; they successfully estimated only N. However, the ability of this method to monitor crop N status in the field has been significantly enhanced by recent work analysing leaves of rice [26], corn [27] and cotton [28]. The same situation is for the SAP test that have been developed to measure nutrient concentrations in a number of vegetable crops including potato [29], tomato [30], cabbage [31], cauliflower [32] and capsicum [33]. Both SPAD chlorophyll meter and SAP test are inexpensive and give rapid results which accuracy mainly depends on type, variety and phenological stage of the cultivation. Times of collection of petioles during the day for the SAP test are important if SAP nutrient concentrations show diurnal variation observed in beets [34] and also in tomato plants [35]. In addition, while plant N concentration declines with crop biomass accumulation, the N concentration per unit leaf area within the upper layer of canopy would remain more or less constant [12]; moreover, a vertical gradient in the canopy N concentration can be observed [36]. So the SAP test may be not able to show a decrease in plant N accumulation during the crop cycle since petioles are collected anytime at the top of the plants. Although many other factors can affect petiole nitrate concentration such as cultivar, temperature and solar radiation [37], the petiole SAP showed to be a reliable diagnostic tool for about 2/3 of the crop cycle (*i.e.*, until the end of linear growth phase) when it is really important for N fertilizer management in processing tomato.

The tomato crop analyzed in this work resulted in a deficiency N concentration phase comparing with the critical N curve presented by Tei *et al.* [9], especially until the early flowering period. Therefore, the estimation efficiency of the N concentration of tomato leaves determined by the SPAD and the SAP test, considering also the separate sampling period, was always underperforming respect to what indicated in literature. This fact could probably depend on the crop deficiency condition in terms of N concentration, referring to the critical N curve [9]. The limited availability in the sample of elements with a concentration of leaf N exceeding critical thresholds may have limited the predictive power of both tests (SPAD and SAP).

In this study the utilization of the portable VIS-NIR spectrophotometer, increasing efficiency, allows better knowledge of nutritional status of tomato plants, with more detailed and sharp information and on wider areas. More detailed information either in space (increase in detail) and in time (the system allowed to perform spectral measurements with an acquisition time of 2 s per leaf, for 500–800 leaves and 100–150 plants) is an essential tool to increase and stabilize crop quality levels and to optimize the nutrient use efficiency, mainly in low input production models.

## Figures and Tables

**Table 1.** List of the different X and Y pre-processing techniques applied in the analysis.

**Label**	**Description**
None	No pre-processing
Log 1/R	Transformation of reflectance in absorbance following log(1/R) formula
Diff1	differences between adjacent variables (approximate derivatives)
Log10	Log 10
logdecay	Log Decay Scaling
Baseline	Baseline (Weighted Least Squares)
Abs	Takes the absolute value of the data
Autoscale	Centres columns to zero mean and scales to unit variance
Detrend	Remove a linear trend
GLS Weighting	Generalized Least Squares Weighting
Groupscale	Group/block scaling
mean centre	Centre columns to have zero mean
msc (mean)	Multiplicative scatter correction with offset, the mean is the reference spectrum
median centre	Centre columns to have zero median
Normalize	Normalization of the rows
Osc	Orthogonal Signal Correction
Sg	Savitsky-Golay smoothing and derivatives
Snv	Standard Normal Deviate
Centering	Multiway Center
Scaling	Multiway Scale
sqmnsc	Scale each variable by the square root of its mean

**Table 2.** Results of linear regression prediction of N concentration in tomato leaves from SPAD (chlorophyll meter readings) and SAP (measurements of N-NO_3_ concentration in petiole) analysis. Efficiency parameters reported: correlation coefficient (*r*), Standard Error of Prediction (SEP), Root Mean Square Error (RMSE), Squared bias (SB), nonunity (NU) and lack of correlation (LC).

**Parameter**	**SPAD**	**SAP**

**MODEL (85%)**		

n° samples	100	100
*r* (measured *vs.* predicted)	0.5383	0.5638
SEP	0.6260	0.6135
RMSE	0.3918	0.3763

**TEST (15%)**		

n° samples	17	17
*r* (measured *vs.* predicted)	0.5589	0.5594
SEP	0.7169	0.7537
RMSE	0.6420	0.6978
SB	0.12	0.12
NU	0.0003	0.0517
LC	0.4834	0.483

**Table 3.** Results of Partial Least Squares (PLS) multivariate analysis on the four different datasets (W = whole dataset, 125 vars; R1 = restricted dataset, first 92 vars, 400–694 nm; R2 = restricted dataset, 46 vars, 400–694 nm, represented by the mean values of each single consecutive pair of steps; R3 = restricted dataset, 62 vars, 496–694 nm) predicting the N concentration in tomato leaves from spectral reflectance analysis. In the table are reported: number of Latent Vectors (LV), Root mean square error in calibration (RMSEC) and validation (RMSECV), correlation coefficient (*r*), Standard Error of Prediction (SEP) and Root Mean Squares Error (RMSE).

	**W**	**R1**	**R2**	**R3**

n° total samples	117	117	117	117
n° LV	4	8	11	11
First pre-processing X-block	Log1/R	None	Log1/R	Log1/R
Second pre-processing X-block	sg	snv	sg	snv
Pre-processing Y-block	none	autoscale	autoscale	none
RMSEC	0.4942	0.5744	0.6079	0.4294
RMSECV	0.5120	0.7990	0.7924	0.5700

**MODEL (85%)**				

n° samples	100	100	100	100
*r* (measured *vs.* predicted)	0.7436	0.8165	0.7917	0.8134
SEP	0.4967	0.4308	0.4533	0.4316
RMSE	0.4942	0.4286	0.4510	0.4294

**TEST (15%)**				

n° samples	17	17	17	17
*r* (measured *vs.* predicted)	0.8856	0.8921	0.9244	0.9414
SEP	0.4186	0.4255	0.3597	0.3466
RMSE	0.4271	0.6460	0.5997	0.4054

## References

[1] Agostini F, Tei F, Silgram M, Farneselli M, Benincasa P, Aller MF, Lichtfouse E (2010). Decreasing nitrate leaching in vegetable crops through improvements in N fertiliser management. Genetic Engineering, Biofertilisation, Soil Quality and Organic Farming.

[2] Farneselli M, Benincasa P, Tei F Validation of N nutritional status tools for processing tomato.

[3] Singandhupe RB, Rao GGSN, Patil NG, Brahmanand PS (2003). Fertigation studies and irrigation scheduling in drip irrigation system in tomato crop (*Lycopersicon esculentum* L.). Eur. J. Agron.

[4] Lemaire G Diagnostic Tool (s) for Plant and Crop N Status. Theory and Practice for Crop N Management.

[5] Sandoval-Villa M, Wood CW, Guertal EA (2002). Tomato leaf chlorophyll meter readings as affected by variety, nitrogen form, and nighttime nutrient solution strength. J. Plant Nutr.

[6] Gianquinto G, Sambo P, Borsato D (2006). Determination of SPAD threshold values in order to optimise the nitrogen supply in processing tomato. Acta Hort.

[7] Monje OA, Bugbee B (1992). Inherent limitations of nondestructive chlorophyll meters: A comparison of two types of meters. HortScience.

[8] Coulombe J, Villeneuve S, Belec C, Tremblay N (1999). Evaluation of soil and petiole sap nitrate quick tests for broccoli in Quebec. Acta Hort.

[9] Tei F, Benincasa P, Guiducci M (2002). Critical nitrogen concentration in processing tomato. Eur. J. Agron.

[10] Jimenez S, Ales JI, Lao MT, Plaza B, Perez M (2006). Evaluation of nitrate quick tests to improve fertigation management. Commun. Soil Sci. Plant Anal.

[11] Hartz TK (2003). The assessment of soil and crop nutrient status in the development of efficient fertilizer recommendations. Acta Hort.

[12] Lemaire G, Gastal F, Lemaire G (1997). N uptake and distribution in plant canopies. Diagnosis of nitrogen status in crops. Diagnosis of Nitrogen Status in Crops.

[13] Menesatti P, Antonucci F, Pallottino F, Roccuzzo G, Allegra M, Stagno F, Intrigliolo F (2010). Estimation of plant nutritional status by Vis-NIR spectrophotometric analysis on orange leaves [*Citrus sinensis* (L) Osbeck cv Tarocco]. Biosyst. Eng.

[14] Benincasa P, Tosti G, Boldrini A, Tei F, Guiducci M Poliannual Results on Soil N Management and Maize N Nutrition by Green Manuring.

[15] Benincasa P, Tosti G, Tei F, Guiducci M (2010). Actual N availability from winter catch crops used for green manuring in maize cultivation. J. Sustain. Agr.

[16] Isaac RA, Johnson WC (1976). Determination of total nitrogen in plant tissue, using a block digestor. J. AOAC.

[17] Harrop Galvao RK, Ugulino Araujo MC, Emıdio Jose G, Coelho Pontes MJ, Cirino Silva E, Bezerra Saldanha TC (2005). A method for calibration and validation subset partitioning. Talanta.

[18] Wold S, Sjostrom M, Erikssonn L (2001). PLS-regression: A basic tool of chemometrics. Chemometr. Intell. Lab. Syst.

[19] Gauch HG, Hwang JTG, Fick GW (2003). Model evaluation by comparison of model-based predictions and measured values. Agron. J.

[20] Waskon RM, Westfall DG, Spellman DE, Soltanpour PN (1996). Monitoring nitrogen status of corn with a portable chlorophyll meter. Commun. Soil Sci. Plant Anal.

[21] Guimarães TG, Fontes PCR, Pereira PRG, Alvarez V, Monnerat PH (1996). Análise de NO_3__ na matéria seca e determinação rápida de NO_3__ na seiva do pecíolo com microeletrodo portátil na avaliação do status nitrogenado do tomateiro. Hortic. Brasil.

[22] Gausman HW (1977). Reflectance of leaf components. Remote Sens. Environ.

[23] Yoder BJ, Daley LS (1989). Development of a visible spectroscopic method for determining chlorophyll a and b *in vivo* in leaf samples. Spectroscopy.

[24] Min M, Lee WS, Kim YH, Bucklin RA (2006). Nondestructive detection of nitrogen in chinese cabbage leaves using VIS-NIR spectroscopy. HortScience.

[25] Esposti MDD, Siqueira DLD, Pereira PRG, Venegas VHAL, Salomão LCC, Machado Filho JA (2003). Assessment of nitrogenized nutrition of citrus rootstocks using chlorophyll concentrations in the leaf. J. Plant Nutr.

[26] Turner FT, Jund MF (1991). Chlorophyll meter to predict nitrogen top-dress requirement for semi dwarf rice. Agron. J.

[27] Wood CW, Reeves DW, Duffield RR, Edmisten KL (1992). Field chlorophyll measurements for evaluation of corn nitrogen status. J. Plant Nutr.

[28] Wood CW, Tracy PW, Reeves DW, Edmisten KL (1992). Determination of cotton nitrogen status with a hand-held chlorophyll meter. J. Plant Nutr.

[29] Williams CMJ, Meir NA (1990). Determination of the nitrogen status of irrigated potato crops. I. Critical nutrient ranges for nitrate-nitrogen in petioles. J. Plant Nutr.

[30] Lyons DL, Barnes JA (1987). Field diagnostic test for nitrate in tomato petiole SAP. Qld. J. Agr. Anim. Sci.

[31] Scaife A, Stevens KL (1983). Monitoring sap nitrate in vegetable crops: Comparison of test strips with electrode methods, and effects of time of day and leaf position. Commun. Soil Sci. Plant Anal.

[32] Kubota A, Thompson TL, Doerge TA, Godin RE (1996). A petiole SAP nitrate test for cauliflower. HortScience.

[33] Olsen JK, Lyons DJ (1994). Petiole SAP nitrate is better than total nitrogen in dried leaf for indicating nitrogen status and yield responsiveness of capsicum in subtropical Australia. Aust. J. Exp. Agr.

[34] Minotti PL, Stankey DL (1973). Diurnal variation in nitrate concentrations in beets. HortScience.

[35] Coltman RR (1987). Sampling consideration for nitrate quick test for green house grown tomatoes. J. Am. Soc. Hort. Sci.

[36] Houlès V, Guérif M, Mary B (2007). Elaboration of a nitrogen nutrition indicator for winter wheat based on leaf area index and chlorophyll content for making nitrogen recommendations. Eur. J. Agron.

[37] Justes E, Meynard JM, Mary B, Plénet D, Lemaire G (1997). Diagnosis using stem base extract: JUBIL method. Diagnosis of the Nitrogen Status in Crop.

